# Does COVID-19 vaccination affect risk perception and adherence to preventive behaviors? A systematic review and meta-analysis

**DOI:** 10.3389/fpubh.2025.1661015

**Published:** 2025-11-12

**Authors:** Michele Sorrentino, Claudio Fiorilla, Fabiana Rubba, Paolo Montuori, Raffaele Palladino

**Affiliations:** 1 Department of Public Health, University of Naples Federico II, Naples, Italy; 2 PhD National Program in One Health Approaches to Infectious Diseases and Life Science Research, Department of Public Health, Experimental and Forensic Medicine, University of Pavia, Pavia, Italy; 3 Interdepartmental Research Center in Healthcare Management and Innovation in Healthcare (CIRMIS), Naples, Italy; 4 Department of Primary Care and Public Health, School of Public Health, Imperial College, London, United Kingdom

**Keywords:** COVID-19, risk perception, adherence, preventive behaviors, systematic review, meta-analysis

## Abstract

**Introduction:**

Since the COVID-19 pandemic onset, preventive measures (e.g., social distancing, hand hygiene, mask usage) and vaccines have been pivotal in mitigating transmission and reducing public health burdens. Although adherence to these measures, influenced by factors such as ventilation and exposure duration, has been extensively validated, their long-term sustainability faces socio-economic challenges.

**Objectives:**

To investigate the association between risk perception and adherence to preventive behaviors and conduct a meta-analysis comparing these behaviors in vaccinated versus unvaccinated subgroups.

**Methods:**

A systematic review following PRISMA guidelines identified studies (2021–2024) analyzing risk perception and preventive behaviors. Potential biases were assessed using the MMAT tool. A meta-analysis calculated pooled effect sizes across subgroups.

**Results:**

Of 1,594 screened studies, 10 met inclusion criteria (six for meta-analysis, *n* = 9,115). Populations included adults, students, and healthcare professionals across 24 countries. Most vaccinated individuals maintained preventive behaviors despite stable or declining risk perception, though social distancing and hand hygiene adherence decreased over time. Booster-vaccinated individuals exhibited higher compliance than partially vaccinated or unvaccinated counterparts. Unvaccinated individuals intending to vaccinate reported higher risk perception than those refusing vaccination. Meta-analysis revealed no significant difference in risk perception between vaccinated (70.3, 95% CI 60.8–79.8) and unvaccinated subgroups (70.8, 95% CI 61.9–79.6; *I*^2^ = 17.5%), suggesting limited influence on behavior maintenance.

**Conclusion:**

While vaccination and preventive measures curbed COVID-19 transmission, risk perception alone does not robustly predict sustained adherence, potentially due to risk compensation. Future research should prioritize determinants of long-term behavioral retention in public health strategies.

## 1 Introduction

Since the World Health Organization (WHO) declared COVID-19 a global pandemic in March 2020 (1), coordinated efforts to limit viral transmission and mitigate strain on public health systems have been implemented worldwide (2). Preventive measures, including lockdowns, social distancing, hand hygiene, and mask usage, were widely recognized as effective strategies to reduce transmission (3). Concurrently, the rapid development and deployment of COVID-19 vaccines played a critical role in pandemic control (4).

Airborne transmission risk is modulated by factors such as ventilation quality (5), exposure duration (6), and adherence to preventive measures (7–9). These interventions not only curtailed COVID-19 spread but also reduced the incidence of other airborne diseases, such as seasonal influenza (10, 11). However, long-term adherence to measures like physical distancing has proven challenging due to socio-economic and behavioral barriers (12, 13). Lockdowns and travel restrictions further strained global economies and supply chains (14), underscoring the need for sustainable strategies.

Successful immunization campaigns enabled a gradual return to normalcy (15–17), yet maintaining preventive behaviors remains critical to minimize pathogen transmission and bolster pandemic preparedness (18).

Risk perception—defined as the intuitive assessment of potential hazards—shapes health-related decision-making (19, 20). This cognitive process involves two components: (1) estimating the probability of harm and (2) subjectively evaluating its severity (21). Differently, risk preferences reflect stable tendencies toward risk avoidance or acceptance (22). Both constructs influence health behaviors, including vaccination uptake and adherence to preventive measures (23).

The introduction of COVID-19 vaccines raised questions about their impact on risk perception and behavioral adherence. Vaccinated individuals may exhibit risk compensation; wherein reduced threat perception diminishes protective behaviors (24). While evidence suggests vaccination lowers self-perceived risk and compliance (24), individuals with heightened public health awareness often sustain preventive practices, particularly in high-risk settings (25, 26). Motivation, mediated by risk perception, remains a key predictor of adherence (27), though messaging and individual awareness also play roles (28).

As the success of current and future public health campaigns on reducing the impact of COVID-19 and other infectious diseases dwell on the possibility to improve both vaccination uptake and adherence to non-pharmaceutical intervention. While the implementation of these measures, during the pandemic, was common and widely accepted, the post-pandemic transition has seen a significant drop in both vaccination and adherence to preventive measures (29). However, understanding the dynamic of retaining preventive measures, not only for the purpose of containing the impact of pandemic, but also for the effect on other air-borne transmitted infection is pivotal to prevent their diffusion. Therefore, this systematic review and meta-analysis aim to assess the impact of COVID-19 vaccination on risk perception change as a predictor of sustained preventive behaviors during the COVID-19 pandemic.

## 2 Methods

This systematic review and meta-analysis examined the association between COVID-19 vaccination and risk perception change as a predictor of sustained preventive behaviors during the COVID-19 pandemic. The study was conducted in accordance with the Preferred Reporting Items for Systematic Reviews and Meta-Analyses (PRISMA) guidelines (30).

### 2.1 Eligibility criteria

Eligible studies included original research (e.g., cohort, cross-sectional, case-control, or qualitative studies) addressing risk perception, preventive behaviors, and vaccination status across all ages, genders, races, and socioeconomic groups. Exclusion criteria included non-English publications, reviews, commentaries, and studies lacking primary data. A summary of eligibility criteria is provided in Table 1.

**Table 1:** Eligibility criteria.

Inclusion criteria
Population
Vaccinated
AND
Un-vaccinated individuals
Intervention
COVID-19 vaccination
Outcome
Risk perception
AND/OR
Adherence to preventive behaviors
Other criteria
Written in English
AND
Original research

### 2.2 Search strategy

This systematic review used the PICO framework to define the inclusion criteria as follows: Population (P): individuals of any age, gender, race, or socioeconomic group; Intervention/Exposure (I): COVID-19 vaccination; Comparator (C): not applicable; Outcomes (O): changes in risk perception and sustained preventive behaviors.

A systematic search was conducted in PubMed/MEDLINE, Embase, PsycINFO (EBSCOhost), Health Technology Assessment Database, and Web of Science (Clarivate) from January 2020 to 22 March 2025 using the following Boolean string: (“risk perception”) AND (“COVID*” OR “SARS*”) AND (“Behav*”). Additional studies were identified through manual searches of reference lists from relevant reviews. The correspondence between PICO elements and search terms is reported in Table 2.

**Table 2:** Research string explain for each domain.

Study population (P)	Not applicable
AND	
Intervention (I)	“COVID*” OR “SARS*”
AND	
Comparison (C)	Not applicable
AND	
Outcome (O)	“Risk perception” AND “Behav*”
AND	
Geographical area (S)	Not applicable
AND	
Timeframe (T)	Not applicable

### 2.3 Data extraction and quality assessment

Two reviewers (MS and CF) independently screened titles/abstracts using Rayyan Artificial Intelligence (31), resolving discrepancies through discussion with a senior reviewer (RP). Full texts were reviewed for ambiguous abstracts. Data on study design, population, outcomes, and risk perception metrics were extracted into a standardized Excel template. Study quality was assessed using the Mixed Methods Appraisal Tool (MMAT), revised version (32), which evaluates methodological rigor across five domains (quantitative, qualitative, mixed methods). Scores range from 0% (no criteria met) to 100% (all criteria met). Studies were retained regardless of quality but flagged for sensitivity analysis if scoring below 50%. For mixed-methods studies, the overall score reflected the weakest component.

### 2.4 Statistical analysis

Studies from the systematic review were included in a meta-analysis comparing risk perception between vaccinated and unvaccinated subgroups. Risk perception scores were standardized as percentages [mean/ (scale maximum) × 100]. Subgroups included: Vaccinated: fully vaccinated (four doses), boosted (three doses), or partially vaccinated (1–2 doses); and Unvaccinated: no doses received.

Pooled effect sizes were calculated using random-effects models (DerSimonian-Laird estimator) with inverse-variance weighting. Heterogeneity was assessed via *I*^2^ statistics, and meta-regression explored variance across subgroups. Analyses were performed in Stata (MP 18.0) with the meta and metan packages.

## 3 Results

### 3.1 Study selection

**Figure 1:**
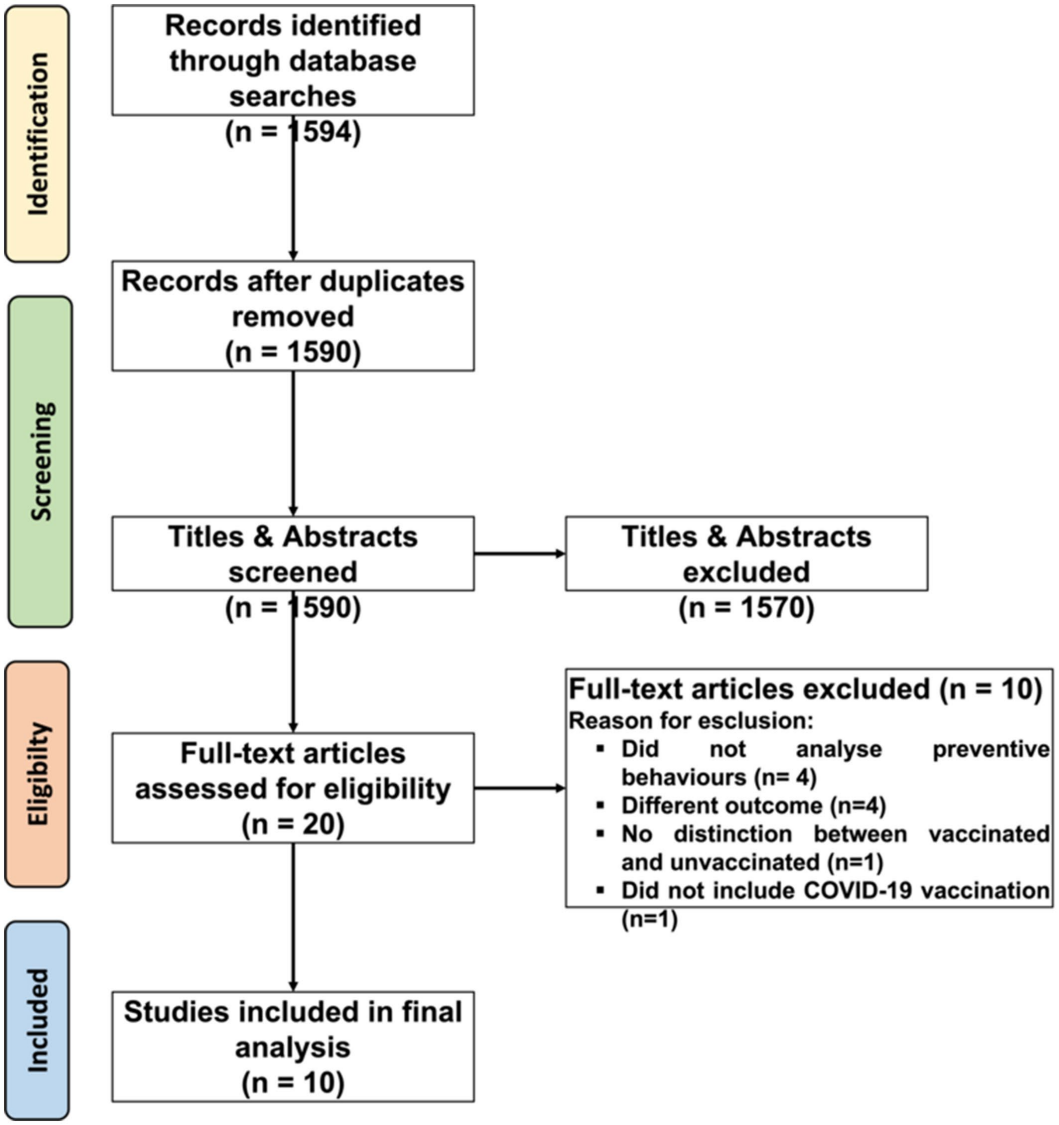
Prisma diagram.

The main research identified 1,594 studies. Following deduplication in Rayyan, 1,590 unique records were retained. Title and abstract screening excluded 1,570 studies, leaving 20 for full-text assessment. Of these, 10 met inclusion criteria, as illustrated in the PRISMA flow diagram (Figure 1). Excluded studies (*n* = 10) were omitted due to: absence of preventive behavior assessments (*n* = 4), irrelevant outcomes (*n* = 4), lack of vaccination status distinction (*n* = 1), or no COVID-19 vaccination focus (*n* = 1). Grey literature, conference papers, dissertations, and editorials were excluded *a priori*.

### 3.2 Study characteristics

The 10 included studies (2021–2024) comprised nine quantitative investigations (six questionnaire-based, three survey-based) and one mixed-methods study (Table 3). Geographically, studies took place in Argentina (*n* = 1), Belgium (*n* = 1), China (*n* = 2), Egypt (*n* = 1), South Korea (*n* = 1), Pakistan (*n* = 1), Saudi Arabia (*n* = 2), the USA (*n* = 1), and 16 European nations (*n* = 1). Study populations focused on general adults (*n* = 6), college/medical students (*n* = 2), healthcare professionals (*n* = 1), and mothers of young children (*n* = 1), with sample sizes ranging from 191 to 221,791 participants.

**Table 3:** Studies characteristics.

First author, year, [cit.]	Country	Study Period	Study design	Method	Population	Sample	Vaccination status	Preventive health behaviors	Risk perception	Quality assessment
Si et al. (37)	China	From March 1st to 21st, 2021	Quantitative study	Questionnaire	Adults	4,540	Yes *n* = 1,825, No *n* = 2,715	Wearing mask, handwashing, keeping physical distancing	Individual health risk perception “The COVID-19 seriously threatens individual health.” Public health risk perception “The COVID-19 seriously threatens public health.”	60%
Qin et al. (34)	China	From June 10 to 15, 2021	Quantitative study	Survey	College students	5,641	All participants vaccinated	Social distancing, mask-wearing, hand washing, sneeze protection, going-out limit, ventilating, and traveling limit	Public health emergency risk perception in 3 domains of dread risk perception, severe risk perception, and unknown risk perception	60%
Torrente et al. (40)	Argentina	March 29th and 30th 2021	Mixed-methods study	Survey	Adults	2,894	Yes *n* = 227, *n* = 2,160 willing to be, *n* = 429 not willing to be, *n* = 78 do not know yet	Use of a mask, physical distancing, and avoidance of enclosed, non-ventilated places	Perceived severity of the disease by the participants in the event of contracting the COVID-19 virus (perceived severity), the perceived likelihood of being infected by the virus (perceived susceptibility), and the current level of fear of the virus (fear of COVID-19)	80%
An et al. (36)	Korea	From 15 October 2021 to 30 October 2021	Quantitative study	Questionnaire	Mothers raising young children under 5 years of age	191	Yes *n* = 160, No *n* = 31 (16.0)	COVID-19 Preventive Health Behaviors	Risk Perception of COVID-19 Infection	60%
Al-Shouli et al. (33)	Saudi Arabia	From 15 September to 11 October 2021	Quantitative study	Questionnaire	Adults	1,010	All participants vaccinated	“I continue to take precautions after receiving COVID-19 vaccine”	“My risk perception toward COVID-19 has increased in comparison to before I received the COVID-19 vaccine”	60%
Hamad et al. (41)	Egypt	From 24 May 2022 to 4 July 2022	Quantitative study	Questionnaire	Medical students	1,884	All participants vaccinated	Keep a safe distance, ensure good ventilation, avoid shaking hands, avoid hugging and kissing cheeks, wear a well-fitting mask, wash hands frequently with soap for 20 s, use antiseptics, avoid crowds, avoid social meetings or events, cover any sneeze in your bent elbow, stay at home when feeling flu-like symptoms, isolate yourself at home if you get in contact with COVID-19 infected patients, eat healthy food, get enough sleep and exercise regularly	Perception of the seriousness of the disease (two items); Extent of anxiety and perception of the susceptibility to the disease (four items); Perceived controllability and self-efficacy of preventive measures (eight items).	80%
Wambua et al. (39)	16 European countries	December 2020–September 2021	Quantitative study	Survey	Adults	29,292	Yes/no	Number of social contacts	“I am likely to catch coronavirus,” “I am worried that I might spread coronavirus to someone who is vulnerable,” “Coronavirus would be a serious illness for me	80%
Waterschoot et al. (38)	Belgium	July 2020–March 2022	Quantitative study	Questionnaire	Adults	221,791	Yes *n* = 76,296, No = 145,495	Handwashing, “to wear your face mask when mandatory or recommended,” and “to maintain physical distance from others.”	Estimated probability to be infected by the coronavirus in the near future and estimated severity of the symptoms when being infected	60%
Liu et al. (23)	USA	June 25–August 24, 2021	Quantitative study	Survey	Adults	1,050	Yes *n* = 72, Planning to *n* = 7, Unsure/Maybe *n* = 8 and Not Planning to *n* = 13	Participation in daily activities and Sum of Mitigation behaviors (SMB) (Maintain social distancing, Wash hands more frequently, Wear gloves away from home, Household cleansing/sanitation, Reduce travel, Wear mask away from home, Use delivery services)	Likelihood of exposure to COVID-19, perceived probability of contracting the virus, willingness to take risk	80%
Chaudhary et al. (35)	Pakistan	From March 10, 2022, to February 25, 2023	Quantitative study	Questionnaire	Medical and dental professionals	410	All participants vaccinated	Mask usage post-vaccination, social distancing post-vaccination, use of sanitizers and frequent hand washing post-vaccination, greetings with a handshake, online shopping instead of going to crowded places like supermarkets post-vaccination, use of public transport post-vaccination	/	100%

Vaccination status categorizations varied: three studies included vaccinated-only cohorts (33–35), four compared vaccinated and unvaccinated individuals (36–39), two stratified by vaccination intent (23, 40), and one differentiated between boosted, partially vaccinated, and unvaccinated subgroups (41). Protective behaviors assessed encompassed mask-wearing, hand hygiene, distancing, and crowd avoidance. Risk perception metrics included personal risk (e.g., perceived severity, infection likelihood) and general risk (e.g., public health impact).

### 3.3 Vaccination status and its impact on risk perception and protective behaviors

Vaccinated individuals predominantly sustained protective behaviors despite static or diminished risk perception. For instance, 62%–78% of vaccinated participants maintained consistent mask use post-vaccination (33, 38, 39), though declines in handwashing (−15%) and distancing (−22%) coincided with increased public transport use (+18%) (35). Notably, boosted individuals demonstrated higher adherence (OR = 1.4, 95% CI 1.1–1.8) than partially or unvaccinated counterparts (41). Unvaccinated individuals intending to vaccinate reported elevated infection risk perception relative to vaccine-refusers (*Δ* = 12.3%, *p* < 0.01) (23). Conversely, no significant behavioral or perceptual differences emerged among mothers of young children regardless of vaccination status (36).

### 3.4 Meta-analysis

**Figure 2:**
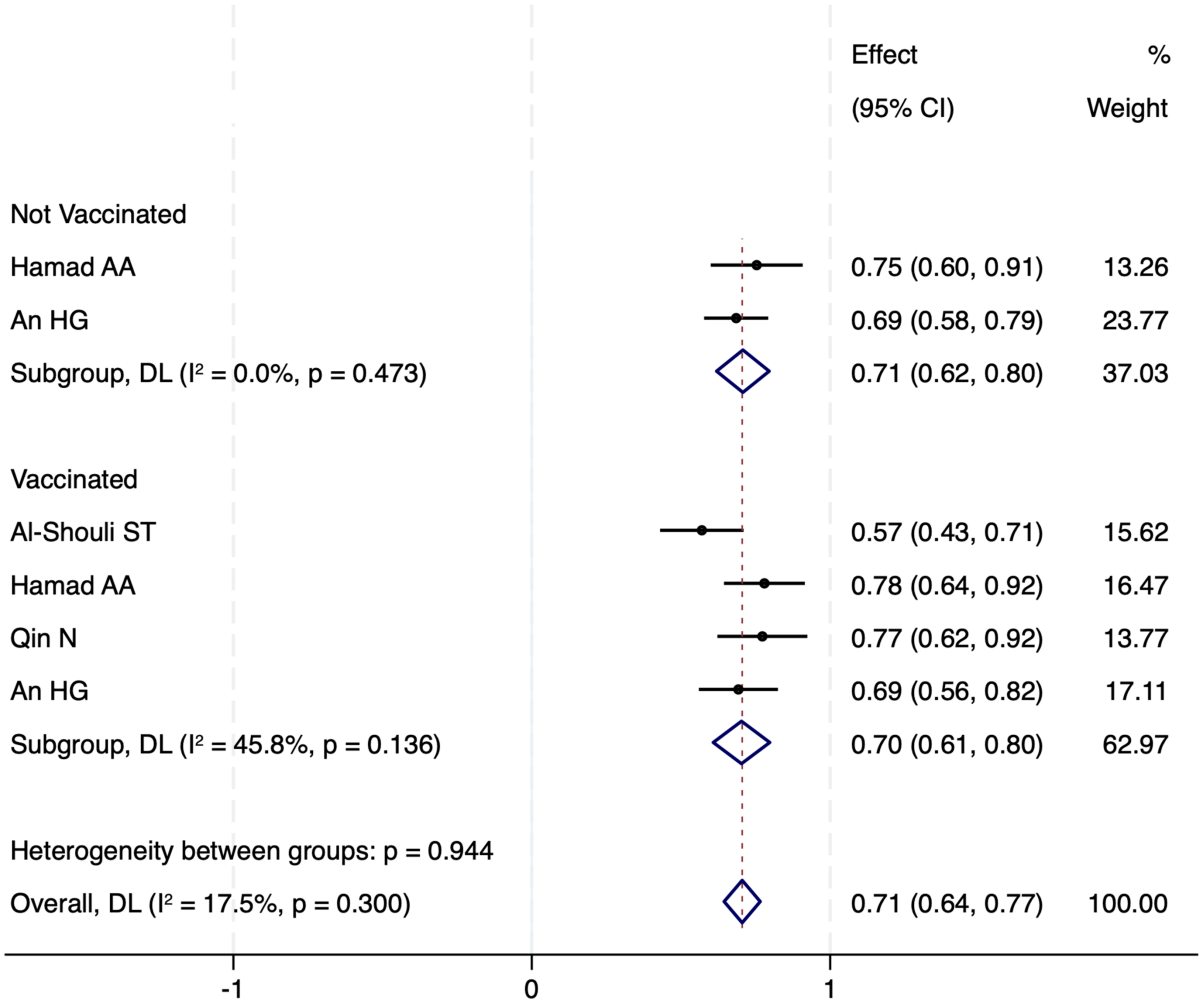
Mentalasisy, all the results are presented as percentage CI are 95%.

Six studies (*n* = 9,115) were included in the meta-analysis after standardizing risk perception scores as percentages (mean/scale maximum × 100). Excluded studies (*n* = 4) lacked comparable risk perception metrics. Pooled estimates revealed minimal differences between vaccinated (70.3, 95% CI 60.8–79.8) and unvaccinated subgroups (70.8, 95% CI 61.9–79.6) (Figure 2). Meta-regression confirmed no significant between-group disparity (*β* = −0.5%, *p* = 0.87), with low heterogeneity (*I*^2^ = 17.5%). A random-effects model corroborated negligible overall variance (*τ*^2^ = 0.02, *Q* = 6.1, *p* = 0.41).

## 4 Discussion

Risk perception and adherence to protective behaviors are pivotal to pandemic management, modulating viral transmission and the efficacy of containment strategies. While COVID-19 vaccination reduced disease severity and transmission, its impact on risk perception and behavioral adherence exhibited marked heterogeneity. Overall, adherence levels remained similar between vaccinated and unvaccinated individuals, with meta-analyses confirming negligible differences and low variance across groups. While some vaccinated individuals sustained protective measures despite static risk perception, others engaged in risk compensation, perceiving vaccination as sufficient protection. These dynamics underscore the need for nuanced public health messaging to promote sustained behavioral adherence.

The 10 studies analyzed—spanning diverse regions, including the USA, China, Belgium, and 16 European nations—reflect varied cultural, social, and political contexts shaping health behaviors. This geographical diversity strengthens generalizability, though it introduces variability in risk perception metrics and behavioral norms.

A significant proportion of vaccinated individuals, maintained precautions post-vaccination, likely driven by persistent awareness of residual risks or social responsibility (33, 34). However, declines in handwashing (−15%) and distancing (−22%) alongside increased public transport use (+18%) (35) suggest risk compensation behaviors (42), wherein perceived vaccine-derived security reduced vigilance. This aligns with evidence that COVID-19 vaccination lowers self-perceived risk and adherence (42–44) underscoring the need to address behavioral complacency in public health campaigns.

Notably, boosted individuals exhibited higher adherence and risk perception than partially/unvaccinated counterparts (41), potentially reflecting heightened awareness of waning immunity. Conversely, unvaccinated individuals intending to vaccinate reported elevated risk perception compared to vaccine refusers (23), suggesting fear of infection and vaccine confidence synergistically drive uptake (45–47).

The meta-analysis revealed no significant difference in risk perception between vaccinated (70.3, 95% CI 60.8–79.8) and unvaccinated subgroups (70.8, 95% CI 61.9–79.6; *β* = −0.5%, *p* = 0.87). This null finding may reflect risk compensation: vaccinated individuals, despite inherently higher baseline risk perception (38, 39), may offset perceived protection via reduced behavioral adherence, attenuating measurable differences between groups. Such compensation is well-documented in health psychology, wherein interventions reducing perceived risk inadvertently disincentivize precautionary behaviors (42–44).

## 5 Policy

Understanding the interplay between COVID-19 vaccination status, risk perception, and protective behaviors is critical for designing adaptive public health strategies. While vaccination campaigns have been pivotal in curtailing disease severity and transmission, their influence on behavioral adherence—marked by risk compensation in some subgroups—demands nuanced policy approaches.

To sustain precautionary measures, initiatives should leverage messaging that: (1) underscores communal responsibility through narratives emphasizing protection of vulnerable populations, as shown in studies linking higher perceived collective risk with greater adherence (48); (2) highlights the persistence of viral evolution, including risks of breakthrough infections and asymptomatic transmission consistent with evidence indicating that awareness of residual risk predicts sustained protective behaviors (49); and (3) addresses risk compensation by reframing vaccination as complementary to—not substitutive for—preventive behaviors. Tailored, culturally resonant communication, disseminated via trusted community leaders and digital platforms, can mitigate complacency while fostering equitable adherence (50).

Integrating these evidence-based insights into policy frameworks will optimize population-level vaccine efficacy and resilience against future pandemics, ensuring public health strategies evolve in tandem with behavioral and epidemiological realities.

## 6 Strengths and limitations

This study offers several strengths. First, it systematically examines the interplay between COVID-19 vaccination status, risk perception, and adherence to preventive behaviors across diverse populations and geographic contexts, enhancing ecological validity. By including both vaccinated and unvaccinated subgroups, it enables nuanced comparisons of behavioral patterns. Adherence to PRISMA guidelines and the integration of meta-analytic methods further bolster methodological rigor, providing a robust quantitative synthesis of global evidence.

However, limitations must be acknowledged. First, the relatively small number of included studies (*n* = 10, of which six were included in the meta-analysis) constrains the external validity of our findings. This limited evidence base may reduce the generalizability of results across different populations, settings, and time periods. To mitigate this, we performed a comprehensive search across multiple databases and applied a rigorous screening process, ensuring that all eligible studies were captured. Further high-quality studies are therefore needed to validate and extend these findings in broader and more diverse cohorts. Second, while the low heterogeneity (*I*^2^ = 17.5%) supports internal consistency, the exclusion of studies with divergent methodologies—though necessary to ensure analytical coherence—may have omitted contextually relevant insights. Nonetheless, sensitivity analyses confirmed that the overall conclusions remained stable despite these exclusions. Third, variations in study design and data collection methods (e.g., self-reported behaviors, cross-sectional frameworks) may have introduced bias, limiting causal inference. To reduce this impact, we carefully assessed study quality and applied consistent inclusion criteria, thereby increasing comparability across studies. Fourth, the predominance of cross-sectional designs precludes longitudinal assessment of risk perception dynamics. Although this limits the ability to infer causality or long-term trends, the convergence of findings across independent studies strengthens confidence in the overall conclusions. Future research should adopt time-series or cohort approaches to expand on these results. Fifth, this review was not prospectively registered in PROSPERO, which may reduce transparency and increase the risk of selective reporting. However, we strictly followed PRISMA guidelines to ensure methodological rigor and transparency, and a complete description of the methodology is provided in the Supplementary material. Finally, restricting the search to English-language studies may have introduced selection bias. This choice, however, is common practice in systematic reviews, as the majority of international scientific literature is conventionally published in English. Moreover, accurate interpretation of findings in multiple foreign languages would have required expertise beyond the scope of the review team. By focusing on English-language studies, we minimized the risk of misinterpretation or inconsistent data extraction, ultimately strengthening the reliability of the synthesis.

## 7 Conclusion

The relationship between COVID-19 vaccination, risk perception, and protective behaviors is shaped by cognitive evaluations, affective responses, and socio-contextual factors. While COVID-19 incidence has markedly declined and preventive measures are no longer widely practiced in many contexts, our findings remain relevant by illustrating how vaccination can interact with risk perception and behavioral adherence. The meta-analytic null finding—no significant difference in risk perception between vaccinated and unvaccinated subgroups—suggests that baseline perceptual differences may be offset by behavioral adjustments post-vaccination.

These insights underscore the necessity of public health strategies that go beyond one-size-fits-all messaging. In future pandemic scenarios or vaccination campaigns for respiratory infections such as influenza or RSV, emphasizing communal responsibility, residual transmission risks, and the complementary role of vaccination and preventive behaviors may mitigate complacency. Moreover, integrating psychological, behavioral, and epidemiological evidence can inform preparedness frameworks and support the design of behaviorally informed interventions. By applying these lessons across infectious threats, policymakers can strengthen resilience, ensure adaptive health strategies, and sustain population-level protection even as epidemiological conditions evolve.

## Data Availability

The original contributions presented in the study are included in the article/Supplementary material, further inquiries can be directed to the corresponding author.
