# Supplementary material for: Does COVID-19 vaccination affect risk perception and adherence to preventive behaviors? A systematic review and meta-analysis
Source: Front Public Health. 2025 Nov 12;13:1661015. doi: 10.3389/fpubh.2025.1661015 (PMC12647121; doi:10.3389/fpubh.2025.1661015)
Supplement: Supplementary file 2 [file Table_2.DOCX]

**Table 1.** *Eligibility criteria*

| Inclusion Criteria |
| --- |
| *Population*  Vaccinated  AND  Un-vaccinated individuals  *Intervention*  COVID-19 vaccination  *Outcome*  Risk perception  AND/OR  Adherence to preventive behaviors  *Other criteria*  Written in English  AND  Original Research |
