# Supplementary material for: Does COVID-19 vaccination affect risk perception and adherence to preventive behaviors? A systematic review and meta-analysis
Source: Front Public Health. 2025 Nov 12;13:1661015. doi: 10.3389/fpubh.2025.1661015 (PMC12647121; doi:10.3389/fpubh.2025.1661015)
Supplement: Supplementary file 3 [file Table_3.DOCX]

**Table 2.** *Research string explain for each domain.*

| Study Population (P) | Not Applicable |
| --- | --- |
| AND | |
| Intervention (I) | "COVID*" OR "SARS*” |
| AND | |
| Comparison (C) | Not applicable |
| AND | |
| Outcome (O) | “risk perception” AND "Behav*" |
| AND | |
| Geographical Area (S) | Not applicable |
| AND | |
| Timeframe   (T) | Not applicable |
