# Supplementary material for: Does COVID-19 vaccination affect risk perception and adherence to preventive behaviors? A systematic review and meta-analysis
Source: Front Public Health. 2025 Nov 12;13:1661015. doi: 10.3389/fpubh.2025.1661015 (PMC12647121; doi:10.3389/fpubh.2025.1661015)
Supplement: Supplementary file 4 [file Table_4.DOCX]

| **First Author, Year, [cit.]** | **Country** |  | **Study Design** | **Method** | **Population** | **Sample** | **Vaccination status** | **Preventive health behaviours** | **Risk perception** | **Quality assessment** |
| --- | --- | --- | --- | --- | --- | --- | --- | --- | --- | --- |
| Si R. et al. , 2021 | China | From March 1st to 21st, 2021 | Quantitative study | Questionnaire | Adults | 4540 | Yes n=1,825, No n=2,715 | Wearing mask, Handwashing, Keeping physical distancing | Individual health risk perception "The COVID-19 seriously threatens individual health."  Public health risk perception "The COVID-19 seriously threatens public health." | 60% |
| Qin N. et al. , 2022 | China | From June 10 to 15, 2021 | Quantitative study | Survey | College students | 5641 | All partecipants vaccinated | Social distancing, mask-wearing, handwashing, sneeze protection, going-out limit, ventilating, and traveling limit | Public health emergency risk perception in 3 domains of dread risk perception, severe risk perception , and unknown risk perception | 60% |
| Torrente F. et al. , 2022 | Argentina | March 29th and 30th 2021 | Mixed-methods study | Survey | Adults | 2894 | Yes n=227, n=2160 willing to be, n=429 not willing to be, n=78 don't know yet | Use of a mask, physical distancing, and avoidance of enclosed, non-ventilated places | Perceived severity of the disease by the participants in the event of contracting the COVID-19 virus (perceived severity), the perceived likelihood of being infected by the virus (perceived susceptibility), and the current level of fear of the virus (fear of COVID-19) | 80% |
| An H.G. et al. , 2022 | Korea | From 15 October 2021 to 30 October 2021 | Quantitative study | Questionnaire | Mothers raising young children under 5 years of age | 191 | Yes n=160, No n=31 (16.0) | COVID-19 Preventive Health Behaviors | Risk Perception of COVID-19 Infection | 60% |
| Al-Shouli S.T. et al. , 2023 | Saudi Arabia | From 15 September to 11 October 2021 | Quantitative study | Questionnaire | Adults | 1010 | All partecipants vaccinated | "I continue to take precautions after receiving COVID-19 vaccine" | "My risk perception towards COVID-19 has increased in comparison to before I received the COVID-19 vaccine" | 60% |
| Hamad A.A. et al. , 2023 | Egypt | From 24 May 2022 to 4 July 2022 | Quantitative study | Questionnaire | Medical students | 1884 | All partecipants vaccinated | Keep a safe distance, Ensure good ventilation, Avoid shaking hands, avoid huggingand kissing cheeks, Wear a well-fitting mask, Wash hands frequently with soap for 20 s, use antispetics, Avoid crowds, avoid social meetings or events, Cover any sneeze in your bent elbow, Stay at home when feeling flu-like symptoms, Isolate yourself at home if you get in contact with COVID-19 infected patients, Eat healthy food, get enough sleep and exercise regularly | Perception of the seriousness of the disease (two items); Extent of anxiety and perception of the susceptibility to the disease (four items); Perceived controllability and self-efficacy of preventive measures (eight items). | 80% |
| Wambua J. et al. , 2023 | 16 European countries | December 2020 - September 2021 | Quantitative study | Survey | Adults | 29,292 | Yes/no | Number of social contacts | "I am likely to catch coronavirus”, “I am worried that I might spread coronavirus to someone who is vulnerable”, “Coronavirus would be a serious illness for me | 80% |
| Waterschoot J. et al. , 2024 | Belgium | July 2020 - March 2022 | Quantitative study | Questionnaire | Adults | 221791 | Yes n=76296, No=145495 | Handwashing, “to wear your face mask when mandatory or recommended”, and “to maintain physical distance from others.” | Estimated probability to be infected by the coronavirus in the near future amd estimated severity of the symptoms when being infected | 60% |
| Liu J. et al. , 2024 | USA | June 25 - August 24, 2021 | Quantitative study | Survey | Adults | 1050 | Yes n=72, Planning to n=7, Unsure/Maybe n=8 and Not Planning to n=13 | Participation in daily activities and Sum of Mitigation Behaviours (SMB) (Maintain social distancing, Wash hands more frequently, Wear gloves away from home, Household cleansing/sanitation, Reduce travel, Wear mask away from home, Use delivery services) | Likelihood of exposure to COVID-19, perceived probability of contracting the virus, willingness to take risk | 80% |
| Chaudhary F.A. et al. , 2024 | Pakistan | From March 10, 2022, to February 25, 2023 | Quantitative study | Questionnaire | Medical and dental professionals | 410 | All partecipants vaccinated | Mask usage post-vaccination, social distancing post-vaccination, use of sanitizers and frequent hand washing post-vaccination, greetings with a handshake, online shopping instead of going to crowded places like supermarkets post-vaccination, use of public transport post-vaccination | / | 100% |

**Table 3.** *Studies characteristics*
